# Appearances after Death in a Case of Hooping-Cough

**Published:** 1824-03

**Authors:** John Webster

**Affiliations:** the Royal College of Physicians of London; Physician to the Royal Infirmary for Sick Children, &c.


Art. IV.—*Appearances after Death in a Case of Hooping-Cough.*

By John Webster, m.d. of the Royal College of Physicians of London; Physician to the Royal Infirmary for Sick Children, &c.

In Nos. 286 and 289 of this Journal, the attention of medical men was directed more particularly to that affection of the head so commonly observed in hooping-cough; and at the same time a new method of treatment was detailed, which had proved very successful; but how far the opinions then expressed relative to the probable seat of the disease may be correct, remains still to be proved by additional experience and observation. However, with a view of increasing the number of facts on this point, it may be useful to give an account of the appearances met with on dissection in the following case; the only fatal one that has come under the author's immediate observation since the publication of the papers already alluded to, although since that period he has had an opportunity of treating 184 cases at the Infirmary for Sick Children alone, according to the method formerly recommended,—namely, the application of leeches to the forehead and behind the ears, blisters, and occasional purgatives.

24th January, 1824.—Frances Willis, ætat. nine months, has been ill of hooping-cough about a fortnight, and has attacks of the disease generally every five or ten minutes, which are exceedingly severe, and exhaust her very much; she tosses her head about when lying down, and appears to suffer greatly in that organ; the eyes look red and heavy, and she is generally very drowsy; on applying the hand to the head, it always feels very hot, accompanied with considerable constitutional fever; pulse accelerated. She feels great oppression at the chest after a fit, which is sometimes followed by expectoration, but never in large quantity. The bowels are rather confined, for which occasional purgatives have been given; but the appetite to suck is not much impaired. Two leeches were applied to the forehead, and a purge of calomel and jalap was prescribed. Three days afterwards, the child felt better, and the fits of hooping were not so frequent, though still very troublesome; but the patient appeared more weak and exhausted, and, when asleep, the eyes were always a little open; and the urine scanty and high-coloured. From the exhausted state of the infant, it was not deemed prudent to repeat the leeches, &c.: therefore, calomel, extract of conium, and squills, were prescribed three times a-day; and a mixture containing nitrous ether, with ipecacuanha wine, was given. Other remedies were afterwards administered, but all without effect, and in fourteen days from admission the patient died, having every symptom of hydrocephalus, with occasional fits of hooping, but not so severe as at first.

On opening the chest, about thirty hours after death, the external appearance of the lungs was quite healthy; no adhesions were found in any part of the thorax; when pressed between the fingers and thumb, the lungs had quite that emphysematous feel peculiar to healthy structure; and there was no induration or hepatization in any part of them. The larynx and trachea were slit open with a pair of scissars; and the ramifications of the bronchia were minutely and carefully traced into the different lobes of the lungs, but no diseased appearance whatever was observed at any point, except at the bifurcation of the trachea, where there was a slight blush of redness on its internal surface, with a small collection of frothy, but healthy mucus. There was no abrasion of surface or unusual vascularity: indeed, it scarcely seemed different from the natural state, and the cut surfaces of any portion of the lungs, when squeezed, did not furnish large spots of mucus at the minute open ends of the bronchia. The heart was quite natural in appearance, and no water was contained in the pericardium. The abdominal viscera were healthy.

After the scull-cap was sawn through, considerable difficulty was encountered in detaching it from the dura mater, which adhered firmly to its internal surface. When the brain was exposed, great vascularity was observed on both hemispheres; the convolutions were so pressed together as almost to make them disappear, it was necessary to press rather firmly with the handle of the scalpel in order to separate them from each other. The pia mater was very vascular, and there was a good deal of effusion of a serous-like fluid under it, especially at the anterior and upper part of the brain, where a few spots of coagulated lymph were seen; the two hemispheres were also slightly adhering to each other at the anterior part, by means of small shreds, like minute vessels, emanating from the pia mater. In the ventricles, about two ounces of serum were found: on the internal surface of these cavities, a number of small vessels, filled with blood, were remarked ramifying on it, particularly on the lower part or floor; and the choroid plexus on the left side was rather larger than usual. The cerebellum was healthy; but the vascularity observed on the superior part of the brain extended also to the basis, and, in the sheath of the medulla oblongata, nearly half an ounce of fluid was collected.

No comment is offered on the above case: it is now published, as well in support of what has been already advanced, as with the view of inducing others to give the result of their experience; so that, by the accumulation of evidence and actual observation, what is really the pathology of hooping-cough may be established on the basis of morbid anatomy.

*56, Grosvenor-street; Feb. 14th, 1824.*

